# Latin American Study of Hereditary Breast and Ovarian Cancer *LACAM*: A Genomic Epidemiology Approach

**DOI:** 10.3389/fonc.2019.01429

**Published:** 2019-12-20

**Authors:** Javier Oliver, Rosalía Quezada Urban, Claudia Alejandra Franco Cortés, Clara Estela Díaz Velásquez, Ana Lorena Montealegre Paez, Rafael Adrián Pacheco-Orozco, Carlos Castro Rojas, Reggie García-Robles, Juan Javier López Rivera, Sandra Gaitán Chaparro, Ana Milena Gómez, Fernando Suarez Obando, Gustavo Giraldo, Maria Isabel Maya, Paula Hurtado-Villa, Ana Isabel Sanchez, Norma Serrano, Ana Isabel Orduz Galvis, Sandra Aruachan, Johanna Nuñez Castillo, Cecilia Frecha, Cecilia Riggi, Federico Jauk, Eva María Gómez García, Claudia Lorena Carranza, Vanessa Zamora, Gabriela Torres Mejía, Isabelle Romieu, Carlos Arturo Castañeda, Miluska Castillo, Rina Gitler, Adriana Antoniano, Ernesto Rojas Jiménez, Luis Enrique Romero Cruz, Fernando Vallejo Lecuona, Iván Delgado Enciso, Abril Bernardette Martínez Rizo, Alejandro Flores Carranza, Verónica Benites Godinez, Claudia Fabiola Méndez Catalá, Luis Alonso Herrera, Yolanda Irasema Chirino, Luis Ignacio Terrazas, Sandra Perdomo, Felipe Vaca Paniagua

**Affiliations:** ^1^Medical Oncology Service, Hospitales Universitarios Regional y Virgen de la Victoria, Institute of Biomedical Research in Malaga, CIMES, University of Málaga, Málaga, Spain; ^2^Laboratorio de Secuenciación, Instituto de Medicina Traslacional e Ingeniería Biomédica, Hospital Italiano de Buenos Aires, Buenos Aires, Argentina; ^3^Laboratorio Nacional en Salud, Diagnóstico Molecular y Efecto Ambiental en Enfermedades Crónico-Degenerativas, Facultad de Estudios Superiores Iztacala, Tlalnepantla de Baz, Mexico; ^4^Unidad de Biomedicina, Facultad de Estudios Superiores Iztacala, UNAM, Tlalnepantla de Baz, Mexico; ^5^Instituto de Nutrición, Genética y Metabolismo, Facultad de Medicina, Universidad El Bosque, Bogota, Colombia; ^6^Grupo INPAC, Organización Keralty, Departamento de Genética, Clínica Universitaria Colombia, Bogotá, Colombia; ^7^Grupo INPAC, Organización Keralty, Facultad de Medicina, Fundación Universitaria Sanitas, Bogotá, Colombia; ^8^Servicio de Genética, Hospital Universitario San Ignacio, Bogotá, Colombia; ^9^Instituto de Genética Humana, Facultad de Medicina, Pontificia Universidad Javeriana, Bogotá, Colombia; ^10^Clínica Universitaria Bolivariana, Pontificia Universidad Bolivariana, Medellín, Colombia; ^11^Departamento Ciencias Básicas de Salud, Facultad de Ciencias de la Salud, Pontificia Universidad Javeriana Cali, Cali, Colombia; ^12^Centro Médico Imbanaco, Cali, Colombia; ^13^Departamento Materno Infantil, Facultad de Ciencias de la Salud, Pontificia Universidad Javeriana Cali, Cali, Colombia; ^14^Fundación Cardiovascular de Colombia, Centro de Investigaciones, Floridablanca, Colombia; ^15^Departamento de Investigación y Estudios Clínicos, IMAT - Oncomédica S.A., Montería, Colombia; ^16^Instituto de Medicina Traslacional e Ingeniería Biomédica, CONICET-Instituto Universitario del Hospital Italiano-Hospital Italiano de Buenos Aires, Buenos Aires, Argentina; ^17^Servicio de Ginecología, Hospital Italiano de Buenos Aires, Buenos Aires, Argentina; ^18^Centro Oncológico Estatal ISSEMyM, Toluca de Lerdo, Mexico; ^19^INVEGEM, Guatemala, Guatemala; ^20^Instituto Nacional de Salud Pública, Cuernavaca, Mexico; ^21^Hubert Department of Global Health, Emory University, Atlanta, GA, United States; ^22^Departamento de Oncología Médica, Instituto Nacional de Enfermedades Neoplásicas, Lima, Peru; ^23^Departamento de Investigación, Instituto Nacional de Enfermedades Neoplásicas, Lima, Peru; ^24^Fundación Alma, Ciudad de México, Mexico; ^25^Instituto Estatal de Cancerología de Colima, Colima, Mexico; ^26^Laboratorio de Investigación Biomédica, Unidad Académica de Medicina, Universidad Autónoma de Nayarit, Tepic, Mexico; ^27^Instituto Mexicano del Seguro Social, Ciudad de México, Mexico; ^28^Unidad de Investigación Biomédica en Cáncer, Instituto de Investigaciones Biomédicas-Instituto Nacional de Cancerología, Ciudad de México, Mexico; ^29^Departamento de Patología, Hospital Universitario Fundación Santa Fe de Bogotá, Bogota, Colombia; ^30^Instituto Nacional de Cancerología, Ciudad de México, Mexico

**Keywords:** breast cancer susceptibility, massively parallel sequencing, germline pathogenic variants, Latin America, HBOC

## Abstract

**Purpose:** Hereditary Breast and Ovarian Cancer (HBOC) syndrome is responsible for ~5–10% of all diagnosed breast and ovarian cancers. Breast cancer is the most common malignancy and the leading cause of cancer-related mortality among women in Latin America (LA). The main objective of this study was to develop a comprehensive understanding of the genomic epidemiology of HBOC throughout the establishment of The Latin American consortium for HBOC-LACAM, consisting of specialists from 5 countries in LA and the description of the genomic results from the first phase of the study.

**Methods:** We have recruited 403 individuals that fulfilled the criteria for HBOC from 11 health institutions of Argentina, Colombia, Guatemala, Mexico and Peru. A pilot cohort of 222 individuals was analyzed by NGS gene panels. One hundred forty-three genes were selected on the basis of their putative role in susceptibility to different hereditary cancers. Libraries were sequenced in MiSeq (Illumina, Inc.) and PGM (Ion Torrent-Thermo Fisher Scientific) platforms.

**Results:** The overall prevalence of pathogenic variants was 17% (38/222); the distribution spanned 14 genes and varied by country. The highest relative prevalence of pathogenic variants was found in patients from Argentina (25%, 14/57), followed by Mexico (18%, 12/68), Guatemala (16%, 3/19), and Colombia (13%, 10/78). Pathogenic variants were found in *BRCA1* (20%) and *BRCA2* (29%) genes. Pathogenic variants were found in other 12 genes, including high and moderate risk genes such as *MSH2, MSH6, MUTYH*, and *PALB2*. Additional pathogenic variants were found in HBOC unrelated genes such as *DCLRE1C, WRN, PDE11A*, and *PDGFB*.

**Conclusion:** In this first phase of the project, we recruited 403 individuals and evaluated the germline genetic alterations in an initial cohort of 222 patients among 4 countries. Our data show for the first time in LA the distribution of pathogenic variants in a broad set of cancer susceptibility genes in HBOC. Even though we used extended gene panels, there was still a high proportion of patients without any detectable pathogenic variant, which emphasizes the larger, unexplored genetic nature of the disease in these populations.

## Introduction

Breast and ovarian cancers have been recognized as heterogeneous diseases comprising several molecular subtypes, each one with an associated risk profile and specific treatment recommendations (1–3). The majority of the cases are sporadic; however, 5–10% of breast and ovarian cancers occur in patients with germline predisposition variants (4). Patients predisposed to breast and ovarian cancer have the phenotype that is defined as the Hereditary Breast and Ovarian Cancer (HBOC) syndrome.

Latin America (LA) is a region encompassing 33 countries, with diverse culture, geography, and ethnicity. Their resources, health care systems, and socioeconomic status are highly diverse among countries. Genetic studies have shown a unique history of migration to this region, combined with a high diversity of native population per country and important population bottlenecks (5, 6). Thus, LA is considered the most genetically admixed population in the world, and its specific combinations of genetic ancestries may impact on the genomic determinants of diseases (7). In Latin America, breast cancer is the most common malignancy and the leading cause of cancer-related mortality among women (8). By country, the highest standardized incidence rates of breast cancer by age are present in Argentina, Brazil and Uruguay (between 67.7 and 71.9) and the lowest in Bolivia (12.7) and El Salvador (7.9). Geographic variation of breast cancer incidence in LA may be explained by reproductive patterns, lifestyle factors, possibility of early detection and access to healthcare (9) together with ethnicity and different ancestral genetic components (7, 10). In terms of mortality, Uruguay (20.5), Argentina (19.4), and Cuba (14.9) show the higher rates whereas Guatemala (3.9) and El Salvador (4.1) show the lowest (9). Moreover, both incidence and mortality proportions for Latin American women aged under 44 years have been shown to be higher when compared with those found in high income countries (20 vs. 12% and 14 vs. 7%, respectively), suggesting an important contribution in LA of prevalent risk factors and genetic background (11).

Germline variation in *BRCA1* and *BRCA2* (*BRCA1/2*) genes is responsible for up to half of the heritable pathogenic variants in HBOC. However, germline variants in other recognized HBOC susceptibility genes have been identified including *ATM, CDH1, CHEK2, PALB2, PTEN, STK11*, and *TP53* (12). Recently, germline pathogenic variants in other genes present in the same DNA repair pathway as *BRCA1/2* have been identified and are associated with HBOC, including *RAD51C, BRIP1, BARD1*, and *MRE11A* (12, 13). In addition, recent reports have shown a potential implication of pathogenic heterozygotic alterations of genes related to other DNA repair mechanisms such as non-homologous end joining (NHEJ), Fanconi anemia pathway (*FANCM, FANCI, FANCL, FANCC, FANCB, FANCF, FANCE*), base-excision repair (*MUTYH*), nucleotide excision repair (*ERCC2, ERCC6*), and mismatch repair (*MLH1, MLH3, MSH2, MSH5, MSH6*) (12, 14–16). Nevertheless, clinical guidelines for appropriate testing and interventions are not yet available for these genes and pathogenic variants are reported exclusively in the research context.

Currently, there is a limited volume of studies of genetic susceptibility to HBOC in Latin America, and the majority of them are focused on the *BRCA1/2* genes. Only a few publications have described the association of pathogenic variants in genes other than *BRCA1/2* in selected HBOC cases in patients from Colombia (17), Mexico (18), Brazil (19, 20), and Chile (21). Therefore, a network of collaborators and cancer specialists in LA joined common efforts to create a *The Latin American consortium for HBOC*-*LACAM*. The *LACAM* study aims to recruit a large series of 3,000 cases from multiple centers in LA in 4 years, which will make it the largest study of HBOC in these populations. This population size will ensure accurate and robust findings on the role of germline pathogenic variants in cancer susceptibility genes and modifying risk factors. The purpose of this work is to describe the standard procedures for patient recruitment, data collection, sample preparation and next-generation sequencing (NGS) analysis of cancer susceptibility gene panels for the *LACAM* consortium. Additionally, we describe the frequency of germline variations in an initial cohort of 222 individuals who fulfilled the criteria for HBOC from Argentina, Colombia, Guatemala and México using two NGS gene panels.

## Materials and Methods

### *LACAM* Participating Countries and Institutions

We identified countries in the Latin American region with few or absent genomic studies for HBOC. Aiming to cover the diversity of populations of this region in terms of ancestry and sociocultural factors, we included representative countries from North America: Mexico; Central America: Guatemala; and South America: Colombia, Peru; and the Southern cone, including Argentina.

Participants are being recruited at 11 centers, including 2 centers from Estado de Mexico and Mexico City in Mexico (Centro Oncológico Estatal de Toluca, Instituto Mexicano del Seguro Social Siglo XXI), 6 centers from different regions in Colombia (Clínica Universitaria Colombia, Hospital Universitario San Ignacio, UPB Clínica Universitaria Bolivariana, IMAT-Oncomédica S.A., Centro Médico Imbanaco, Fundación cardiovascular de Colombia), one center in Peru (Instituto Nacional de Enfermedades Neoplásicas), Argentina (Hospital Italiano de Buenos Aires) and Guatemala (Instituto para la Investigación Científica y la Educación Acerca de las Enfermedades Genéticas y Metabólicas Humanas). The *LACAM* protocol was approved by the Ethics Committee of each center (INEN-18-06, FM-CIE-0409-17, HI-2730, COE/UEI/PT/02/2018, INSP-CI:1065, INSP-341, CE/INVEGEM 1-2017, UEB.471-2018, ONC-CEI-801-2018, UPB-2018, ISEM 28-09-2015, CEICANCL290515-05GENCMAHER) and it is conducted in accordance with the Declaration of Helsinki.

### *LACAM* Recruitment Protocol and Standard Operating Procedures (SOPs)

All individuals were enrolled based on the criteria established in the Genetic/Familial High-Risk Assessment: Breast and Ovarian of the National Comprehensive Cancer Network (NCCN) guidelines, version 2.2018 (22). Participating individuals were identified in the Clinical genetics, Oncology or Gynecology consultation of each institution (incident cases) or through the review of clinical records (prevalent cases). Prevalent cases were also interviewed to confirm that they met the inclusion criteria. Each study participant received a unique identification number based on the center number and a 4-digit patient number. This code is allocated upon patient's agreement to participate in the study. All patients were provided with written informed consent for the participation in this study.

Individuals underwent an interview and completion of a questionnaire about their lifestyle and basic demographic information was requested. The lifestyle questionnaire is structured to collect the following information: (i) Demographic details (age, sex, ethnic origin, city and place of residence and educational status); (ii) History of tobacco use; (iii) History of alcohol consumption, including types of alcoholic beverages; and (iv) Reproductive factors including parity, contraceptive use, age of menarche, and age of menopause.

In addition, we recorded anthropometric measurements: weight and height at time of recruitment, abdominal perimeter, weight up to 2 years before the interview, as well as weight at the age of 20 years. For all patients diagnosed with cancer, there is an additional clinical questionnaire which gathers all the available information on clinical and histopathological diagnosis and treatment. Study data are collected and managed using REDCap electronic data capture tools hosted at Hospital Universitario San Ignacio. REDCap (Research Electronic Data Capture) is a secure, web-based application designed to support data captured from research studies, therefore providing (1) an intuitive interface for validated data entry; (2) audit trails for tracking data manipulation and export procedures; (3) automated export procedures for seamless data downloads to common statistical packages; and (4) procedures for importing data from external sources (23). In all participating centers, clinical geneticists or health care personnel trained in genetic counseling were involved during recruitment in order to guarantee pre- and post-test communication and guidance.

### Standardization of Sequencing Protocol and Analysis

After completion of the first phase of the study (first year of recruitment), we standardized methods for sample preparation, sequencing, and variant analysis using the first 222 individuals included in this study.

### Sample Preparation

Blood extraction was performed by trained personnel at the time of the interview unless a more convenient time was available. 10-mL samples of blood are collected with EDTA in order to obtain: whole blood, buffy coat (leukocytes/platelets), red blood cells, and plasma. All samples are stored at each center at −80°C. In any case the amount of time between extraction and freezing does not exceed 12 h. When available, H&E slides and FFPE tumor tissue are collected.

### DNA Extraction

Peripheral blood DNA was extracted with the DNeasy Blood & Tissue Kit (Qiagen) and the Magna Pure System (Roche) following the manufacturer's instructions. The DNA concentration was quantified with the Qubit dsDNA HS Assay Kit (Invitrogen) and the integrity and purity of the material was verified by agarose gel electrophoresis and spectrophotometry.

### Preliminary Information on Sequencing Variants

To evaluate the prevalence of germline pathogenic variants in individuals with criteria for hereditary disease from Latin America, we performed NGS of peripheral blood DNA of 68 individuals from Mexico, 57 from Argentina and 19 from Guatemala and analyzed a panel of 143 cancer susceptibility genes. The resulting pathogenic variants were polled with those from 78 Colombian patients sequenced with the Trusight kit (94 cancer susceptibility genes). All pathogenic variants correspond to genes shared by both panels, with the exception those found in *DCLRE1C, MLH3, PDE11A, PDGFB*.

### Library Preparation and Massive Parallel Sequencing

Library preparation and sequencing of the Mexican, Argentinian and Guatemalan samples was performed with the GeneRead Cancer Predisposition V2 Kit (Qiagen), as previously described (18). Briefly, the kit targets 143 genes associated with inherited cancers. The genes have been selected on the basis of their putative role in the susceptibility of different hereditary cancers. Most, but not all, are associated with hereditary breast and ovarian cancer and with an increased risk to 88 oncological syndromes such as colorectal, ovarian, endometrial, prostate, gastric, and pancreatic cancers among others; and almost all these genes have evidence supporting a >2-fold increase in risk. The libraries were sequenced in a MiSeq instrument (Illumina, Inc.; 2X150 cycles) to reach an average theoretical mean coverage of 80X for each sample (Supplementary Figures 1, 2). The DNA samples from Argentina were prepared with Generead Library L kit (Qiagen) and sequenced in a PGM (Ion Torrent-Thermo fisher- Scientific). The libraries from Colombia were prepared using the Trusight cancer panel (Illumina, Inc.), which includes a panel of 96 genes (40 genes overlap with the 143 gene panel) and sequenced in a MiSeq instrument (Illumina, Inc.; 2X150 cycles).

### Sequencing Normalization and Pathogenic Variant Detection

The variant reduction to identify pathogenic variants was done as reported previously (18). Briefly, FastQC files were aligned to the human genome reference hg19 with BWA-MEM; indels were realigned and the bases were recalibrated. Adaptors were soft-clipped and reads with <20 bp were eliminated. Variant calling was done with HaplotypeCaller (Broad Institute) and annotation with ANNOVAR and InterVar (24, 25). Pathogenic variant description was done following Human Genome Variation Society (HGVS) nomenclature (http://www.hgvs.org/) and variant classification followed the five-tier criteria of the American College of Medical Genetics and Genomics (ACMG) (26) and was manually curated. Synonymous variants and those with depth <5X or with mutant allele fraction <20% were excluded. Splicing and null variants (stop-gain/loss, frameshift indels) and missense variants previously defined as pathogenic in ClinVar were considered unequivocally pathogenic (https://www.ncbi.nlm.nih.gov/clinvar). Null variants present at the 3′ end of the gene that were reported as conflicting in ClinVar were classified as “unknown clinical significance” (VUS). Minor allelic frequency <0.001 in the gnomeAD (http://gnomad.broadinstitute.org/) database was used to capture rare, potentially pathogenic, null and missense variants. Low frequency (<0,001) missense variants predicted as deleterious by SIFT or PolyPhen-2 but with no further evidence of *in vitro/vivo* or clinical pathogenicity were classified as VUS. All filtered variants were manually curated by inspection of the BAM files with the IGV software (Broad Institute). All pathogenic and likely pathogenic variants were confirmed experimentally by two independent Sanger sequencing assays. Variants in *BRCA1* and *BRCA2* were further assessed in the Huntsman Cancer Institute Breast Cancer Genes Prior Probabilities site (http://priors.hci.utah.edu/PRIORS/index.php) to evaluate their potential impact. Variants in *MLH1, MSH2, MSH6, PMS2* were also investigated in the Leiden Open Variation Database (http://hci-lovd.hci.utah.edu/home.php). Both pathogenic and likely pathogenic variant categories were grouped as “pathogenic” to simplify presentation and discussion of our results.

### Detection of Exon 9-12 Deletion in BRCA1

The founder mutation consisting in a deletion in exons 9–12 was detected by PCR amplification of the mutant and wild-type alleles, using specific primers based the method published in Weitzel et al. (18, 27). The PCR products were resolved in 1.5% agarose gels to identify the amplification of the truncated allele and sequenced.

### Large Rearrangement (LR) Analysis

Patients from Colombia without *BRCA1* or *BRCA2* pathogenic variants were tested for MLPA (Multiplex Ligation-dependent Probe Amplification), using SALSA® MLPA® probemix P002 BRCA1 and P090 BRCA2 kits.

### Statistical Analysis

Characteristics of all recruited individuals and cases with confirmed diagnosis of cancer were summarized with descriptive statistics. The association between demographic and clinical characteristics on the presence of pathogenic variants was assessed using univariate analyses (unadjusted logistic regression model). Age at recruitment, age at diagnosis for cancer cases and body mass index (BMI) were included as continuous variables, whereas the rest of the factors were considered as categorical variables. The logistic regression model utilized all available data (complete and missing). *p* < 0.05 was considered to indicate statistical significance. All the analyses were conducted using STATA 13.0.

## Results

### Initial LACAM Recruitment in 5 Countries

Four hundred and three individuals have been recruited in the first phase of the *LACAM* study from October 2017 to March 2019 (Supplementary Table 2). Epidemiological characteristics with complete information were obtained for more than 80% of individuals and are listed in Table 1. Fifty-seven percent of individuals had a family history of cancer, 72% reported at least one pregnancy and the average parity was three children (SD: 1.7), 37% never used oral contraceptives and 65% reported not being current or former alcohol consumers or smokers. Importantly, 48% percent of all individuals were overweight or obese at time of recruitment.

**Table 1:** Epidemiological characteristics of the 403 individuals recruited in the first phase of the *LACAM* study.

**Epidemiological Characteristics (*****N*** **Total** **=** **403)**					
	***N***	**%**		***N***	**%**
**Countries**			**Smoking history**		
Argentina	57	14.1	Never smoker	252	65.5
Colombia	160	39.7	Former smoker	60	14.9
Guatemala	19	4.7	Current smoker	10	2.5
Mexico	116	28.8	No Information	81	20.1
Peru	51	12.7	**Alcohol history**		
BMI			Never drinker	263	65.3
Underweight (<18.5)	8	2.0	Former drinker	34	8.4
Normal (18.5 <25)	155	38.4	Current drinker	25	6.2
Overweight (25.0 <30)	128	31.8	No Information	81	20.1
Obese (>30)	64	15.9	**Family history of cancer**		
Missing	48	11.9	Yes	230	57.1
Age, y			No	90	22.3
18–45	190	47.2	Missing	83	20.6
46–60	162	40.2	**Pregnancy**		
61–70	30	7.4	Yes	292	72.5
≥71	16	4.0	No	81	20.1
Missing	5	1.2	Missing	30	7.4
Median age (range)	46	(20–82)	**Median age 1st pregnancy (range)**	23	(15–41)
Gender			**Median pregnancies (range)**	2	(1–10)
Male	9	2.2	**Median months of breastfeeding (range)**	14	(1–72)
Female	394	97.8	**Median age menarche (range)**	12	(6–20)
Race/ethnicity			**Ever use of oral contraceptives**		
White	119	29.5	Yes	167	41.4
Black	1	0.3	No	149	37.0
Mestizo/Mulatto	194	48.1	Missing	87	21.6
Asiatic	1	0.3	**Cancer diagnosis**		
Indigenous	6	1.5	Yes	356	88.3
Other/unknown	82	20.3	No	47	11.7
Education level					
Post graduate	27	6.7			
Graduate	89	22.1			
Superior-Technical	80	19.9			
Secondary	75	18.6			
Primary	45	11.2			
None	3	0.7			
No information	84	20.8			

Eighty-eight percent of the individuals had a cancer diagnosis from which 75% corresponded to breast cancer cases (Table 2). Complete staging (pathological or clinical) information was available for 42% of cases.

**Table 2:** Clinical characteristics of 356 cancer patients recruited in the first phase of the *LACAM* study.

**Clinical Characteristics (*****N*** **=** **356)**		
	***N***	**%**
**PRIMARY CANCER SITE**		
Breast	268	75.3
Ovary	75	21.1
Multiple primaries (*)	4	1.1
Missing	9	2.5
**HISTOPATHOLOGICAL SUBTYPE BREAST CANCER**		
DCIS	1	0.4
IDC	173	64.5
ILC	13	4.9
MC	1	0.4
TN	8	3.0
Missing	72	26.9
**HISTOPATHOLOGICAL SUBTYPE OVARIAN CANCER**		
SC	46	61.3
EC	5	6.7
CCC	3	4.0
Missing	21	28.0
**STAGE AT DIAGNOSIS**		
I	25	7.0
II	53	14.9
III	68	19.1
IV	4	1.1
No Information	206	57.9

DCIS, Ductal carcinoma in situ; IDC, Invasive ductal carcinoma; ILC, Invasive lobular carcinoma; MC, Medullary carcinoma; TN, Triple negative; SC, Serous carcinoma; EC, Endometrioid carcinoma; CCC, Clear cell carcinoma. (^*^) Breast, Colon, Thyroid, Pancreas, Endometrial.

### Preliminary Sequencing Results

**Figure 1:**
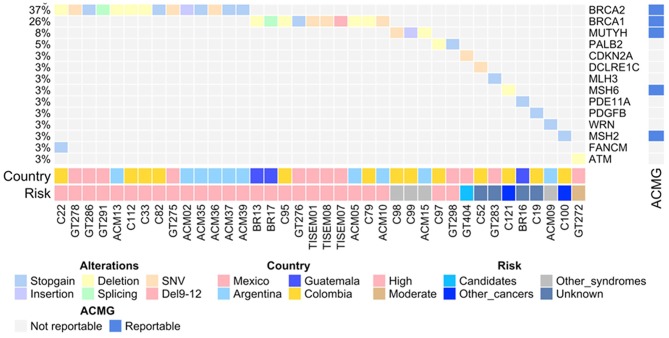
The OncoPrint shows the allelic distribution of the pathogenic variants in patients with cancer. The grid panel depicts the pathogenic mutations found in each patient color-coded for each type. Right panel: gene reportable by the suggestion of the ACMG (blue: yes, gray: no). Bottom axis: patient ID. Left axis: relative frequency of mutations per gene. Right axis: mutated gene. Right bar plot: absolute frequency and type of pathogenic mutation per gene. Bottom panel indicates: Country; risk associated with the variant.

Initial sequencing results on 222 individuals showed that the overall prevalence of individuals with pathogenic variants was 17% (38/222) and the distribution spanned 14 genes and varied by country (Figure 1, Table 3). The highest relative prevalence of pathogenic variants was found in patients from Argentina (25%, 14/57), followed by Mexico (18%, 12/68), Guatemala (16%, 3/19), and Colombia (13%, 10/78). Twenty-five percent (1/4) of the pathogenic variants found in *BRCA1* in the Mexican patients corresponded to the founder mutation ex9-12del. All pathogenic variants were heterozygous. The overall prevalence of patients with a negative result (without any detectable pathogenic variants or VUS) was 48% (106/222). By country the prevalence of patients with a negative result was Guatemala 53% (10/19), México 52% (36/68), Colombia 47% (37/78), Argentina 40% (23/57). No additional large rearrangements (LR) were found in the 33 individuals from Colombia.

**Table 3:** Pathogenic variants detected in 222 HBOC individuals from 4 countries.

**ID**	**Country**	**Gene**	**Type of change**	**Transcript**	**Exon**	**cDNA change**	**Protein change**
ACM02	ARG	*BRCA2*	Frameshift insertion	NM_000059	22	c.8857_8858insTA	p.E2953fs
ACM05	ARG	*BRCA1*	Frameshift deletion	NM_007294	10	c.3706_3707del	p.N1236fs
ACM09	ARG	*WRN*	Stopgain	NM_000553	9	c.C1105T	p.R369X
ACM10	ARG	*BRCA1*	nsSNV	NM_007294	4	c.A211G	p.R71G
ACM13	ARG	*BRCA2*	Frameshift deletion	NM_000059	10	c.1760delC	p.T587fs
ACM15	ARG	*MUTYH*	Frameshift deletion	NM_001128425	12	c.1147delC	p.L383fs
ACM35	ARG	*BRCA2*	Stopgain	NM_000059	11	c.C2095T	p.Q699X
ACM36	ARG	*BRCA2*	nsSNV	NM_000059	2	c.T2G	p.M1R
ACM37	ARG	*BRCA2*	Stopgain	NM_000059	11	c.G3922T	p.E1308X
ACM39	ARG	*BRCA2*	Stopgain	NM_000059	11	c.G3922T	p.E1308X
BR13	GUA	*BRCA1*	Frameshift deletion	NM_007294	10	c.798_799del	p.V266fs
BR16	GUA	*PDE11A*	Stopgain	NM_016953	2	c.C985T	p.R329X
BR17	GUA	*BRCA1*	Splicing	NM_007294	4	c.G212+1A	–
C100	COL	*MSH2*	Stopgain	NM_000251	7	c.C1165T	p.R389X
C112	COL	*BRCA2*	Frameshift deletion	NM_000059	10	c.1761_1764del	p.T587fs
C121	COL	*MSH6*	Frameshift deletion	NM_000179	5	c.3254delC	p.T1085fs
C19	COL	*PDGFB*	Stopgain	NM_002608	4	c.C445T	p.R149X
C22	COL	*BRCA2*	Frameshift deletion	NM_000059	9	c.696delT	p.Y232fs
C22	COL	*FANCM*	Stopgain	NM_020937	8	c.1360_1361insAACAAAGTTA	p.E455Qfs*2
C33	COL	*BRCA2*	Frameshift deletion	NM_000059	11	c.2806_2809del	p.K936fs
C52	COL	*DCLRE1C*	nsSNV	NM_001033855	6	c.G403A	p.G135R
C79^a^	COL	*BRCA1*	Frameshift deletion	NM_007294	10	c.3331_3334del	p.Q1111fs
C82	COL	*BRCA2*	Stopgain	NM_000059	22	c.C8951A	p.S2984X
C95	COL	*BRCA1*	Frameshift deletion	NM_007294	10	c.1674delA	p.K558fs
C97	COL	*PALB2*	Frameshift deletion	NM_024675	5	c.2288_2291del	p.L763fs
C98	COL	*MUTYH*	nsSNV	NM_001128425	7	c.A536G	p.Y179C
C99	COL	*MUTYH*	Frameshift insertion	NM_001128425	13	c.1228_1229insGG	p.E410fs
GT272	MEX	*ATM*	Frameshift deletion	NM_000051	62	c.8872_8873del	p.F2958fs
GT275	MEX	*BRCA2*	nsSNV	NM_000059	19	c.C8420T	p.S2807L
GT276	MEX	*BRCA1*	Stopgain	NM_007294	12	c.C4327T	p.R1443X
GT278	MEX	*BRCA2*	nsSNV	NM_000059	13	c.G7007A	p.R2336H
GT283	MEX	*MLH3*	Stopgain	NM_001040108	2	c.G82T	p.E28X
GT286	MEX	*BRCA2*	Stopgain	NM_000059	22	c.G8839T	p.E2947X
GT291	MEX	*BRCA2*	Splicing	NM_000059	19	c.G8487+1A	NA
GT298	MEX	*PALB2*	Stopgain	NM_024675	12	c.C3256T	p.R1086X
GT404	MEX	*CDKN2A*	nsSNV	NM_000077	1	c.T146C	p.I49T
TISEM01	MEX	*BRCA1*	nsSNV	NM_007294	17	c.C5123A	p.A1708E
TISEM07^a^	MEX	*BRCA1*	Large deletion	NM_007294	9-12	–	–
TISEM08	MEX	*BRCA1*	Frameshift deletion	NM_007294	7	c.496delA	p.R166fs

*ARG, Argentina; COL, Colombia; MEX, Mexico; GUA, Guatemala; nsSNV, nonsynonymous single-nucleotide variation*.

a *Reported founder mutation*.

Forty eight percent (18/38) of pathogenic variants were found in patients diagnosed with breast cancer, 24% (9/38) in ovarian cancer and 21% (8/38) in individuals without cancer diagnosis but with family history of cancer under HBOC criteria.

#### Pathogenic Variants in BRCA1 and BRCA2 Genes

Twenty percent of the total number of pathogenic variants were found in *BRCA1* and 29% in *BRCA2* genes in the four countries. Noteworthy, patients from Guatemala and Argentina had the highest country proportion of *BRCA1* (40%) and *BRCA2* (71%) pathogenic variants, respectively (Figure 1). Only two previously reported founder mutations were found in two patients from Mexico and Colombia. Finally, all of the patients had one monoallelic pathogenic variant, excluding patient C22 from Colombia, who had two monoallelic variants in *BRCA2* and in *FANCM* (Figure 1, Table 3).

#### Pathogenic Variants in Non-BRCA Genes

Pathogenic variants in non-BRCA genes were found in 12 genes, including those in other high and moderate HBOC risk genes such as *MSH2, MSH6, MUTYH*, and *PALB2*. The hereditary melanoma and pancreatic cancer gene *CDKN2A* was found mutated in one patient from Mexico. Only 3 of these genes, however, are currently suggested as reportable by the ACMG (26) (Table 4). Among the countries included in this study, Colombian individuals were the most affected with pathogenic variants in non-BRCA genes presenting 8 out of 14 participants with these alterations (Table 4). Moreover, pathogenic variants were found in the DNA repair genes *DCLRE1C* (NHEJ) and *WRN* (homologous recombination), along with the cyclic-AMP regulator *PDE11A* in a patient from Guatemala and the growth-promoting gene *PDGFB* in a Colombian patient. Pathogenic variants in non-BRCA genes were more frequent in patients diagnosed with Breast cancer (21%) compared to those with ovarian cancer (11%).

**Table 4:** Hereditary syndromes associated with the pathogenic variants detected in 222 individuals.

**Gene**	**Associated syndrome (mode of inheritance)**	**Frequency of pathogenic variants (proportion of pathogenic variants)**	**Countries with patients affected (country proportion)**	**Signaling pathway**	**Gene reportable by ACMG (5/14)**
ATM	Ataxia-telangiectasia (AR), breast cancer susceptibility (AD)	2.6% (1/39)	MEX (1/12)	Cell cycle, DNA repair	No
BRCA1	Breast cancer susceptibility (AD)	25.6% (10/39)	ARG (2/10); COL (2/14); GUA (2/3); MEX (4/12)	HR	Yes
BRCA2	Breast cancer susceptibility (AD)	35.9% (14/39)	ARG (6/10); COL (4/14); MEX (4/12)	HR	Yes
CDKN2A	Melanoma and neural system tumor syndrome (AD), pancreatic cancer/melanoma syndrome (AD)	2.6% (1/39)	MEX (1/12)	Cell cycle	No
DCLRE1C	Omenn syndrome (AR); severe combined immunodeficiency, athabascan type (AR)	2.6% (1/39)	COL (1/14)	Non-homologous end-joining	No
FANCM	Premature ovarian failure 15 (AR); spermatogenic failure 28 (AR)	2.6% (1/39)	COL (1/14)	Fanconi anemia pathway	No
MLH3	Colorectal cancer, hereditary nonpolyposis, type 7; endometrial cancer susceptibility	2.6% (1/39)	MEX (1/12)	Mismatch repair	No
MSH2	Colorectal cancer, hereditary non-polyposis, type 1 (AD); mismatch repair cancer syndrome (AR); Muir-Torre syndrome (AD)	2.6% (1/39)	COL (1/14)	Mismatch repair	Yes
MSH6	Colorectal cancer, hereditary non-polyposis, type 5 (AD); mismatch repair cancer syndrome (AR); familial endometrial cancer	2.6% (1/39)	COL (1/14)	Mismatch repair	Yes
MUTYH	Multiple colorectal adenomas (AR)	7.7% (3/39)	ARG (1/10)	Base excision repair	Yes
PALB2	Fanconi anemia, complementation group N; breast cancer susceptibility (AD); pancreatic cancer susceptibility	5.1% (2/39)	ARG (1/10); COL (2/14)	Fanconi anemia pathway	No
PDE11A	Pigmented nodular adrenocortical disease, primary, 2 (AD)	2.6% (1/39)	GUA (1/3)	Hydrolysis of cAMP and cGMP, Metabolism of purines	No
PDGFB	Familial susceptibility to meningioma (AD)	2.6% (1/39)	COL (1/14)	Cell proliferation	No
WRN	Werner syndrome (AR)	2.6% (1/39)	ARG (1/10)	DNA replication, HR	No

*AR, autosomal recessive; AD, autosomal dominant; X, X-linked; ARG, Argentina; COL, Colombia; GUA, Guatemala; MEX, Mexico; ACMG, American College of Medical Genetics and Genomics; HR, Homologous recombination*.

Of note, no association was found between epidemiological or clinical characteristics described in Tables 1, 2 and presence of pathogenic alterations in any of the 143 genes evaluated.

#### Variants With Unknown Clinical Significance (VUS)

We detected a total number of 132 VUS representing a prevalence of 41% (90/222). These variants were distributed in 76 genes, including high-risk HBOC genes (*BRCA1, BRCA2, APC, CDKN2A, CDH1, MSH2, MSH6)*, moderate risk (*ATM, CHEK2, RAD51C, PALB2*) and genes with no risk defined to date (Supplementary Table 1). The gene carrying the highest number of VUS was *PMS2*, probably due to equivocal amplification and alignment of the *PMS2* pseudogenes. The following genes with the highest number of VUS were *FANCM* (6/132) *BRCA1* (5/132) *APC* (4/132) and *BRCA2* (4/132). Notably, the proportion of the VUS in non-BRCA genes was 93.2%. Eight percent of individuals (19/222) presented more than one VUS.

## Discussion

Breast cancer is a highly prevalent disease in Latin America (LA) and worldwide (28). The epidemiological transition along with the adoption of unhealthy western life-styles together with changing reproductive factors have influenced the increased incidence of breast cancer in LA (9). Several countries of the LA region have acknowledged this as a public health problem and have specific policies to progress in early detection, which is the most effective measure to control this disease.

The *LACAM* study pursued the primary aim of constructing a network of collaborators to investigate the genetic susceptibility to breast cancer in Latin America focusing mainly on HBOC. We gathered a group of researchers from Argentina, Colombia, Guatemala, Peru and Mexico to recruit individuals from families that fulfill criteria of HBOC. In this first phase of the project we have recruited 403 individuals from five countries, following uniformed procedures for sampling, questionnaire assessment and methods for sequencing analysis.

Fortunately, there has been progress in the region regarding description of HBOC—associated pathogenic variants in the *BRCA1* and *BRCA2* genes. Relevant works have identified pathogenic recurrent founder mutations in Mexico, Colombia, Argentina, Brazil, Chile, Costa Rica, Cuba and Peru (29). Currently, there are few studies reporting causal pathogenic variants in a more complete set of high and moderate HBOC risk genes in the region (17–21), in comparison with dozens of gene panel studies performed in European and American Caucasian populations. This clearly shows the important disparity in the knowledge of the genetic diversity of this disease as compared with other populations (30). Although the contributions made in LA are of major relevance, much effort still needs to be done for the correct identification of the genetic component for to HBOC susceptibility in this region.

We analyzed the genetic profile using two panels of cancer susceptibility genes by next-generation sequencing and we found that 17% (38/222) of the patients carried pathogenic variants in 14 susceptibility genes. Overall, the prevalence of pathogenic variants in the *BRCA1* and *BRCA2* genes in Argentina, Mexico, Guatemala and Colombia reached 14.3, 11.8, 10.5, and 8.1%, respectively. Only two previously reported founder mutations in the *BRCA1* gene were found (18, 27, 29, 31–33): the ex9-12del founder mutation corresponding to 25% (1/4) of the *BRCA1* pathogenic variants found in Mexican patients and the *BRCA1* p.Q1111fs mutation found in a Colombian patient.

Although the number of patients with a pathogenic variant was relatively modest (38/222), the total proportion of pathogenic variants in *BRCA1/2* as compared with other genes was high, 61.5 vs. 38.5%. Indeed, in Colombia, the proportion of pathogenic variants in non-*BRCA* genes was the highest, 57.1% (8/14). Moreover, the fact that 39% of the pathogenic variants were found in 12 genes distinct from *BRCA1/2* genes, highlights the important locus heterogeneity of this disease that is present in these Latin American populations. Importantly, this is the first report of pathogenic variants in non-*BRCA* genes in patients with HBOC for Guatemala and Argentina.

On the other hand, the genes with pathogenic variants associated with other syndromes included the Fanconi Anemia *FANCM*, Lynch syndrome *MLH3, MSH2, MSH6*; the colorectal adenoma *MUTYH*, the pancreatic and melanoma *CDKN2A*; and the ataxia telangiectasia gene *ATM*, as previously reported (15, 34–37).

Pathogenic variants were also detected in genes not frequently associated with HBOC, such as *DCLRE1C* (16), *PDGFB, WRN* (16), and *PDE11A* (18). The *DCLRE1C gene* (cytoband 10p13) codes for the ubiquitously expressed nuclear protein Artemis, which has 5-3′ exonuclease activity and participates in V(D)J recombination and in non-homologous end joining DNA repair, especially in double-strand breaks caused by radiation and oxidative stress (38–41). Germline alterations in this gene have been reported in patients with autosomal recessive conditions such as the Athabascan-type severe combined immunodeficiency (SCIDA) and the Omenn syndrome (42, 43). Artemis is a substrate of phosphorylation by *ATM* in response to DNA damage and interacts with *BRCA1* and the MRN complex composed of MRE11, RAD50, and NBN (44). The pathogenic variant detected in *DCLRE1C*, p.G135R, is located in the b-lactamase domain, which is the catalytic core for V(D)J recombination (45) and has been classified as a probably pathogenic in ClinVar. The intimate role of this protein in DNA repair is highly suggestive of a cancer susceptibility gene when it is altered in heterozygosity. As for the *PDGFB* gene (22q13.1), it codes for a potent mitogen protein that participates in the regulation of multiple processes including embryonic development, cell proliferation, cell migration, survival and chemotaxis. The alteration detected in *PDGFB* (p.R134X) truncates the protein in the N-terminal PDGF/VEGF domain, which is essential for the homo- or heterodimerization with PDGFA and for binding and activation of the PDGFR receptors. Germinal alterations in this gene cause familial susceptibility to meningiomas and brain calcifications, both with an autosomal dominant inheritance pattern (46–48). Particularly, there are no previous reports associating this gene to HBOC susceptibility. The individual carrying this variant (C19) has not been diagnosed with BC, but her family history included a maternal aunt diagnosed with premenopausal breast cancer, a sister with BC at 41 years of age and her mother was diagnosed with lung cancer. It is possible that additional genetic factors or specific exposures could play a role in the aggregation of the disease in this patient and her family.

In a previous study on HBOC we reported *WRN* and *PDE11A* alterations in two Mexican patients (18). In the current analysis, pathogenic variants in these genes we also found in cases from Argentina and Guatemala. The helicase WRN participates in the maintenance of genome stability, by interacting with translesion polymerases to prevent collapse of stalled replication forks (49). The WRN protein also interacts with the Ku70/80 helicases to facilitate DNA repair by the NHEJ pathway (50). Alterations in this gene in homozygosity cause Werner syndrome, characterized by premature aging, but monoallelic pathogenic variants in this gene have been previously reported in patients with inherited breast cancer, and its involvement in genome stability is compatible with a potential susceptibility role (16, 18, 51). On the other hand, *PDE11A* codes for a phosphodiesterase that catalyze the hydrolysis of intracellular concentrations of cyclic AMP and GMP being a negative regulator of growth promoting transduction pathways. Rare germline alterations in *PDE11A* have been associated with prostate and testicular germ cell tumor susceptibility (52, 53). Of importance, the same pathogenic variant detected in *PDE11A*, p.R329X, was identified in our previous study in one Mexican patient (18).

Noteworthy, only 5 out of the 14 genes with pathogenic variants described in our study are suggested to be reported as clinically relevant to the patient by the ACMG (26). Therefore, the overwhelming existing evidence shows that the contribution of new susceptibility genes to inherited breast cancer has been underestimated. In this regard, an effort to better establish the role of these non-*BRCA* genes to HBOC susceptibility in populations from LA is being carried out by the *LACAM* project and will be discussed in future publications.

Of note, in 48% of individuals there was no evidence of a potential disease causal variant, either VUS or pathogenic. These results show there is a large, unexplored genetic landscape of this disease, known as missing heritability (54). We acknowledge that a fraction of the patients could also be affected by large rearrangements (LR) that were not evaluated in individuals from Argentina and Guatemala. On the contrary, patients from Colombia were negative for LR and patients from Mexico were evaluated for the most common large deletion, the *BRCA1* ex9-12del, which comprises almost half of the large rearrangements of this gene in Mexican population. In 2012 Judkins et al. reported that the percentage of LR positivity for Latin American/Caribbean cases in the US who met the criteria for BRCA testing was 6,7% and the ex9-12del of *BRCA1* was 45% (44/97) among these cases (31). Using this percentage and considering that a major proportion of the here presented cohort is of Mexican origin, the number of missed LR in our preliminary analysis is not expected to be high. Additional considerations for negative sequencing results in any of the evaluated genes are the presence of variants in non-coding regions, not assessed, and the possibility that common low-risk variation could be acting in some of these families in combinatorial, epistatic ways. The contribution of the low-risk SNPs and the prevalence of LR will be addressed in the following phase of the *LACAM* project in a larger number of patients.

Unfortunately, the limited number of individuals carrying a pathogenic variant (N = 38) did not allow us to find statistically significant associations with modifying risk factors such as lifestyle and reproductive factors. Therefore, a larger analysis is envisioned for the second phase of our project to better account for the effect of these modifying factors in HBOC as they have never been evaluated in LA populations.

Finally, the ultimate purpose of the *LACAM* study is to provide solid and detailed scientific evidence on the molecular epidemiology of HBOC in the LA population in order to improve intervention strategies, prevention and risk management for patients affected by this disease. Current national cancer plans among 14 countries in LA include secondary prevention strategies with an emphasis on prevention, early detection, and opportune treatment (55). Also, several countries in the region have national guidelines recommending genetic counseling and testing in high-risk individuals (56). Identifying individuals with a high risk of developing breast and ovarian cancer among other associated HBOC tumors allows for more effective detection of the disease at earlier stages and enables the application of cancer prevention strategies among family members when risk assessment is provided before disease onset (22, 57). In order to efficiently implement these approaches, major efforts should be focused on: (i) Availability of centralized genetic testing to core facilities with certified laboratories to both ensure their cost-effectiveness and increase the technical and bioinformatic expertise in LA; (ii) creating open access databases of HBOC variants and large-scale cancer genomics data sets derived from similar studies in Latin American populations; (iii) strengthening the genetic counseling services in all countries of the region and increasing the number of training programs in clinical genetics; (iv) developing educational awareness programs for the general population; (v) generating formal analyses of the economic impact of different genetic testing options in LA to evidence real health benefit costs in the Latin American context; and (vi) engaging health policy makers, insurance companies, political and public health institutions and academia to enforce implementation of national cancer plans and country-specific genetic testing guidelines.

## Conclusions

In this work, we established the framework of *The Latin American consortium for HBOC*-*LACAM*, a joint regional effort aimed to analyze the genetic component and modifying risk factors of inherited breast cancer in individuals from Latin America. In this first phase of the project, we recruited 403 individuals from Argentina, Colombia, Guatemala, Mexico and Peru and evaluated the germline genetic alterations in an initial cohort of 222 patients from 4 countries. The overall prevalence of pathogenic variants was 17% (38/222), distributed along 14 genes. Even if extended gene panels were used, there was still a high proportion of patients without detectable pathogenic variants, which emphasizes the large, unexplored genetic nature of the disease in these populations. The establishment of a possible associated risk of HBOC with non-*BRCA* genes still requires additional scientific evidence taking into consideration ancestral components, common genetic variation and non-genetic variables such as lifestyle and other risk factors. These factors will be addressed in the following phases of the *LACAM* project in a larger number of patients.

## Data Availability Statement

The raw data supporting the conclusions of this manuscript will be made available by the authors, without undue reservation, to any qualified researcher.

## Ethics Statement

The *LACAM* protocol was approved by the Ethics Committee of each center (Instituto Nacional de Enfermedades Neoplásicas INEN-18-06, Hospital Universitario San Ignacio FM-CIE-0409-17, Hospital Italiano de Buenos Aires HI-2730, Centro Oncológico Estatal de Toluca COE/UEI/PT/02/2018, Instituto Mexicano del Seguro Social Siglo XXI INSP-CI:1065, INSP-341, Instituto para la Investigación Científica y la Educación Acerca de las Enfermedades Genéticas y Metabólicas Humanas CE/INVEGEM 1-2017, Universidad El Bosque UEB 471-2018, IMAT-Oncomédica SA ONC-CEI-801-2018, UPB Clínica Universitaria Bolivariana UPB-2018, Instituto de Salud del Estado de Mexico ISEM 28-09-2015, Instituto Estatal De Cancerología De Colima CEICANCL290515-05GENCMAHER) and it is conducted in accordance with the Declaration of Helsinki.

## Author Contributions

AG, EG, CLC, CAC, JO, SP, and FVac: clinical protocol revision. GT, CLC, CAC, CF, EG, AM, and MC: clinical sampling. GT, CLC, CAF, ER, RGi, AA, AM, and MC: questionnaire assessment. RQ, CD, CLC, and CAF: DNA extraction and quality control. RQ, CD, and CAF: NGS Library preparation. JO, CF, LR, RQ, and FVac: bioinformatics analysis. CAF, LR, RQ, JO, SP, and FVac: data curation. CAF, RQ, JO, SP, and FVac: formal analysis and data presentation. RQ, CD, and CM: validation. SP: statistical analysis. CAC, JO, SP, and FVac: funding acquisition. LT, JO, SP, and FVac: resources. JO, SP, and FVac: original draft. SP, JO, and FVac: study conceptualization. All authors are writing, review and editing.

### Conflict of Interest

The authors declare that the research was conducted in the absence of any commercial or financial relationships that could be construed as a potential conflict of interest.

## Supplementary Material

Supplementary Figure 1 Global distribution of the mean sequencing depth. Histogram of sequence depth (X) with density of all sequenced samples. Mean depth is indicated.

Supplementary Figure 2 Distribution of the mean sequencing depth per country. Histogram of sequence depth (X) with density of all sequenced samples by country. Mean depth by country is indicated.

Supplementary Table 1 Variants with unknown clínical significance detected.

Supplementary Table 2 Clinical and epidemiological characteristics of individuals recruited in the first phase of the LACAM study by country. DCIS, Ductal carcinoma *in situ*; IDC, Invasive ductal carcinoma; ILC, Invasive lobular carcinoma; MC, Medullary carcinoma; TN, Triple negative; SC, Serous carcinoma; EC, Endometrioid carcinoma; CCC, Clear cell carcinoma; (^*^) Breast, Colon, Thyroid, Pancreas, Endometrial. NA, Not applicable.
