# Supplementary figures and images for: Latin American Study of Hereditary Breast and Ovarian Cancer LACAM: A Genomic Epidemiology Approach
Source: Front Oncol. 2019 Dec 20;9:1429. doi: 10.3389/fonc.2019.01429 (PMC6933010; doi:10.3389/fonc.2019.01429)

Mean sequence depth

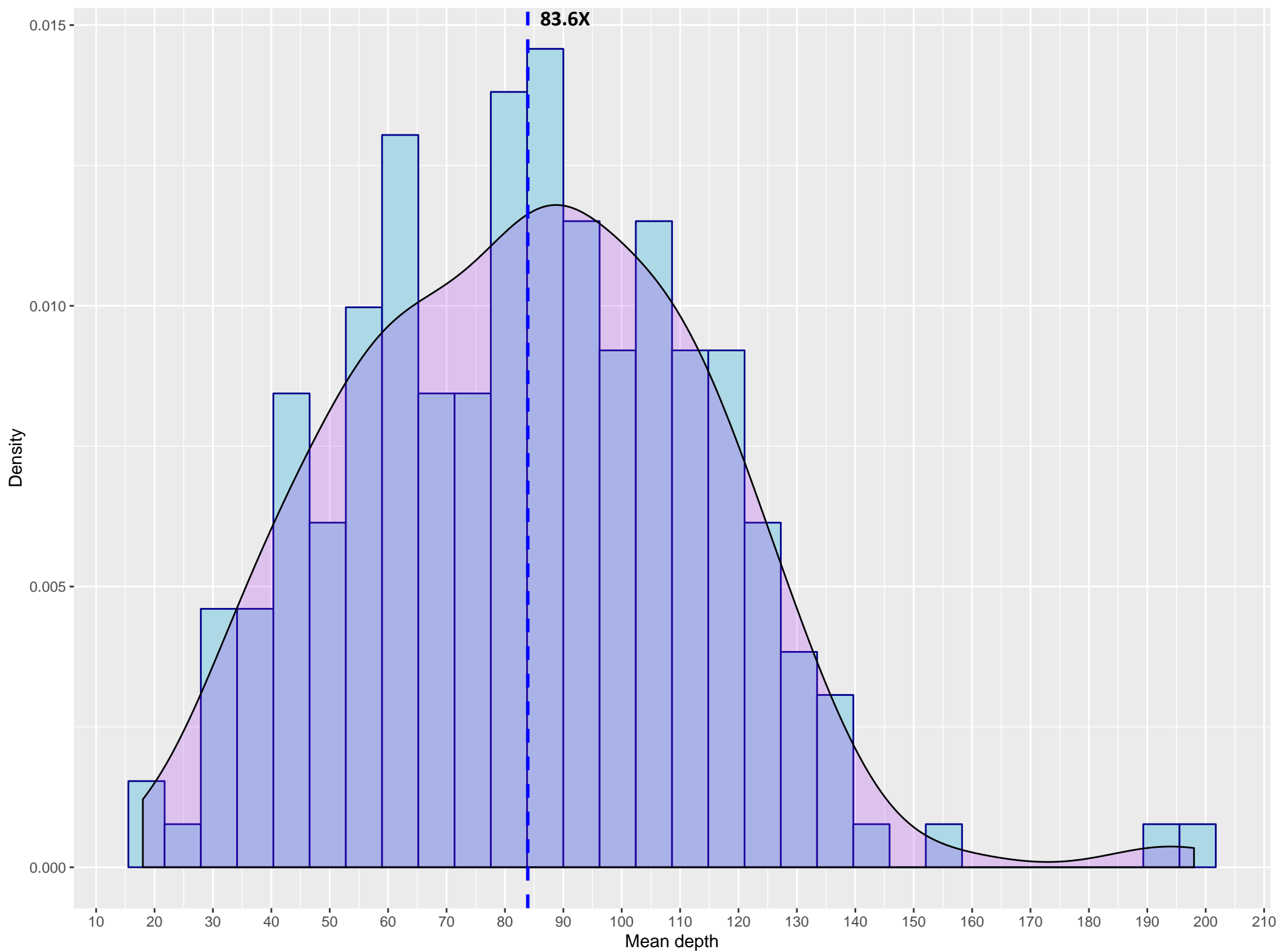

Supplement: Supplementary Figure 1 — Global distribution of the mean sequencing depth. Histogram of sequence depth (X) with density of all sequenced samples. Mean depth is indicated. [file Image_1.PDF]

Mean sequence depth

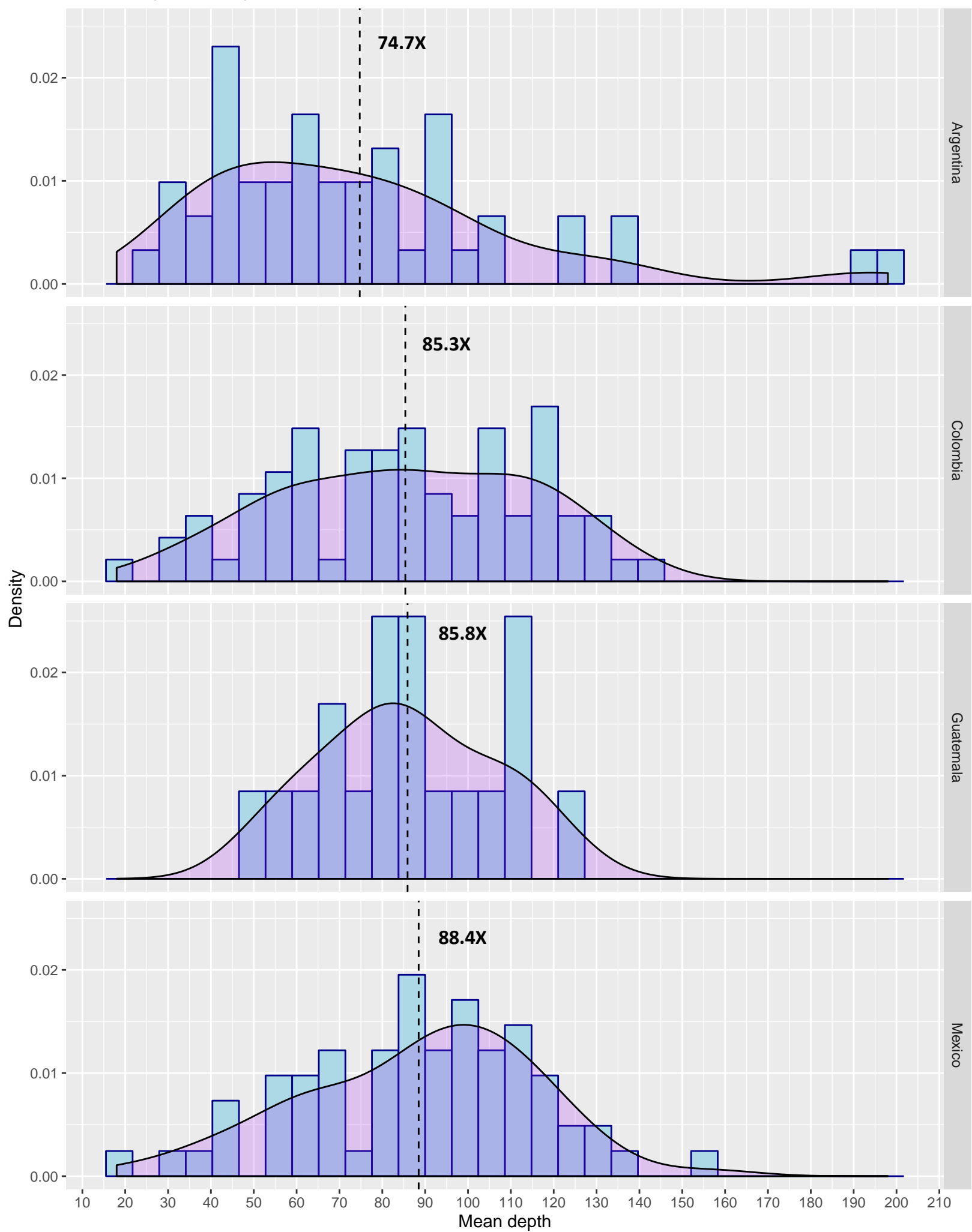

Supplement: Supplementary Figure 2 — Distribution of the mean sequencing depth per country. Histogram of sequence depth (X) with density of all sequenced samples by country. Mean depth by country is indicated. [file Image_2.PDF]
