# Supplementary material for: Latin American Study of Hereditary Breast and Ovarian Cancer LACAM: A Genomic Epidemiology Approach
Source: Front Oncol. 2019 Dec 20;9:1429. doi: 10.3389/fonc.2019.01429 (PMC6933010; doi:10.3389/fonc.2019.01429)
Supplement: Supplementary Table 1 — Variants with unknown clínical significance detected. [file Table_1.DOCX]

**Supplementary Table 1. Variants with unknown clínical significance detected.**

| **ID** | **Country** | **Gene** | **Region** | **Type of change** | **Transcript** | **Exon** | **cDNA change** | **Protein change** |
| --- | --- | --- | --- | --- | --- | --- | --- | --- |
| ACM05 | ARG | *XPC* | exonic | nonsynonymous SNV | NM_004628 | exon4 | c.A535T | p.S179C |
| ACM05 | ARG | *KDR* | exonic | nonsynonymous SNV | NM_002253 | exon16 | c.C2312T | p.T771M |
| ACM05 | ARG | *ATR* | exonic | nonsynonymous SNV | NM_001184 | exon6 | c.G1411C | p.E471Q |
| ACM11 | ARG | *RAD50* | exonic | nonsynonymous SNV | NM_005732 | exon2 | c.G205A | p.D69N |
| ACM13 | ARG | *EPHB2* | exonic | nonsynonymous SNV | NM_001309193 | exon16 | c.C2923G | p.R975G |
| ACM16 | ARG | *TMEM127* | exonic | nonsynonymous SNV | NM_001193304 | exon4 | c.C572T | p.T191M |
| ACM18 | ARG | *PDGFRA* | exonic | nonsynonymous SNV | NM_001347827 | exon11 | c.T1631C | p.V544A |
| ACM26 | ARG | *APC* | exonic | nonsynonymous SNV | NM_000038 | exon16 | c.A5606T | p.D1869V |
| ACM28 | ARG | *ERCC6;PGBD3* | exonic | nonsynonymous SNV | NM_001277059 | exon6 | c.C1790T | p.T597I |
| ACM28 | ARG | *POLE* | exonic | nonsynonymous SNV | NM_006231 | exon15 | c.T1520C | p.V507A |
| ACM29 | ARG | *WRN* | exonic | nonsynonymous SNV | NM_000553 | exon32 | c.C3785G | p.T1262R |
| ACM30 | ARG | *SBDS* | exonic | nonsynonymous SNV | NM_016038 | exon1 | c.T107C | p.V36A |
| ACM31 | ARG | *XPC* | exonic | nonsynonymous SNV | NM_004628 | exon4 | c.G490A | p.E164K |
| ACM31 | ARG | *NBN* | exonic | nonsynonymous SNV | NM_002485 | exon15 | c.C2215G | p.L739V |
| ACM34 | ARG | *MSH6* | exonic | nonsynonymous SNV | NM_000179 | exon4 | c.T1526C | p.V509A |
| ACM34 | ARG | *POLE* | exonic | nonsynonymous SNV | NM_006231 | exon12 | c.C1108A | p.P370T |
| ACM38 | ARG | *BRCA2* | exonic | nonsynonymous SNV | NM_000059 | exon25 | c.A9472G | p.T3158A |
| ACM40 | ARG | *BRCA2* | exonic | stopgain | NM_000059 | exon27 | c.A9976T | p.K3326X |
| ACM41 | ARG | *BRCA1* | exonic | nonsynonymous SNV | NM_007294 | exon19 | c.T5202G | p.F1734L |
| ACM42 | ARG | *PDE11A* | exonic | nonsynonymous SNV | NM_016953 | exon2 | c.G1045A | p.A349T |
| ACM43 | ARG | *SDHA* | exonic | nonsynonymous SNV | NM_004168 | exon10 | c.G1396A | p.A466T |
| ACM43 | ARG | *ERCC2* | exonic | nonsynonymous SNV | NM_000400 | exon5 | c.A284G | p.E95G |
| ACM45 | ARG | *LYST* | exonic | nonsynonymous SNV | NM_000081 | exon26 | c.C7436T | p.A2479V |
| ACM46 | ARG | *BAP1* | exonic | nonsynonymous SNV | NM_004656 | exon5 | c.A358G | p.K120E |
| ACM46 | ARG | *PALLD* | exonic | nonsynonymous SNV | NM_016081 | exon13 | c.T2363G | p.I788S |
| ACM46 | ARG | *AIP* | exonic | nonsynonymous SNV | NM_001302960 | exon1 | c.G26A | p.R9Q |
| ACM48 | ARG | *PTCH1* | exonic | nonsynonymous SNV | NM_000264 | exon6 | c.C926G | p.P309R |
| ACM48 | ARG | *WAS* | exonic | nonsynonymous SNV | NM_000377 | exon6 | c.C538A | p.H180N |
| ACM49 | ARG | *MSR1* | exonic | nonsynonymous SNV | NM_138716 | exon4 | c.A517G | p.I173V |
| ACM50 | ARG | *BARD1* | exonic | nonsynonymous SNV | NM_000465 | exon2 | c.G190A | p.G64R |
| ACM51 | ARG | *RNASEL* | exonic | stopgain | NM_021133 | exon2 | c.G793T | p.E265X |
| ACM51 | ARG | *PTCH1* | exonic | nonsynonymous SNV | NM_000264 | exon22 | c.G3617A | p.R1206H |
| ACM51 | ARG | *CDK4* | exonic | nonsynonymous SNV | NM_000075 | exon4 | c.C458T | p.T153I |
| ACM51 | ARG | *SMARCB1* | exonic | nonsynonymous SNV | NM_003073 | exon6 | c.C749T | p.T250M |
| ACM53 | ARG | *MC1R* | exonic | nonsynonymous SNV | NM_002386 | exon1 | c.C451T | p.R151C |
| ACM54 | ARG | *CD96* | exonic | nonsynonymous SNV | NM_005816 | exon3 | c.C449T | p.T150M |
| ACM54 | ARG | *ATR* | exonic | nonsynonymous SNV | NM_001184 | exon6 | c.G1432C | p.E478Q |
| ACM54 | ARG | *PALLD* | exonic | nonframeshift substitution | NM_001166108 | exon2 | c.671_672CA | p.M224T |
| ACM54 | ARG | *RHBDF2* | exonic | nonsynonymous SNV | NM_001005498 | exon12 | c.A1486G | p.K496E |
| ACM55 | ARG | *FANCL* | exonic | frameshift insertion | NM_001114636 | exon14 | c.1113_1114insAATT | p.T372Nfs*12 |
| ACM56 | ARG | *PALLD* | exonic | nonframeshift substitution | NM_001166108 | exon2 | c.671_672CA | p.M224T |
| ACM56 | ARG | *RAD50* | exonic | nonsynonymous SNV | NM_005732 | exon9 | c.A1277G | p.Q426R |
| BR08 | GUA | *TMC6* | exonic | nonsynonymous SNV | NM_001127198 | exon10 | c.G1193A | p.R398H |
| BR13 | GUA | *PTCH2* | exonic | nonsynonymous SNV | NM_001166292 | exon3 | c.C353A | p.T118N |
| BR14 | GUA | *KIF1B* | exonic | nonsynonymous SNV | NM_183416 | exon12 | c.C1114T | p.R372W |
| BR14 | GUA | *XRCC3* | exonic | nonsynonymous SNV | NM_001100119 | exon8 | c.G623A | p.R208H |
| BR21 | GUA | *EPHB2* | exonic | nonsynonymous SNV | NM_001309193 | exon14 | c.C2540T | p.P847L |
| BR21 | GUA | *ATM* | exonic | nonsynonymous SNV | NM_000051 | exon45 | c.T6493A | p.S2165T |
| BR22 | GUA | *FANCM* | exonic | nonsynonymous SNV | NM_020937 | exon18 | c.G4594C | p.D1532H |
| BR23 | GUA | *PMS2* | exonic | nonsynonymous SNV | NM_000535 | exon2 | c.T124A | p.L42I |
| BR26 | GUA | *MC1R* | exonic | nonsynonymous SNV | NM_002386 | exon1 | c.T949C | p.W317R |
| BR26 | GUA | *ATM* | exonic | nonsynonymous SNV | NM_000051 | exon29 | c.T4267C | p.C1423R |
| C03 | COL | *ADAMTS17* | exonic | nonsynonymous SNV | NM_139057 | exon9 | c.C1317A | p.F439L |
| C03 | COL | *BAG3* | exonic | nonsynonymous SNV | NM_004281 | exon2 | c.C211T | p.R71W |
| C03 | COL | *FRAS1* | exonic | nonsynonymous SNV | NM_025074 | exon9 | c.G886A | p.E296K |
| C04 | COL | *ACAD8* | exonic | nonsynonymous SNV | NM_014384 | exon3 | c.C235T | p.R79W |
| C04 | COL | *PALB2* | exonic | nonsynonymous SNV | NM_024675 | exon13 | c.G3367A | p.V1123M |
| C04 | COL | *PCDH15* | exonic | nonsynonymous SNV | NM_001142763 | exon7 | c.A536G | p.N179S |
| C04 | COL | *GFM1* | exonic | nonsynonymous SNV | NM_001308164 | exon17 | c.C2068T | p.R690C |
| C05 | COL | *CDH1* | exonic | nonsynonymous SNV | NM_004360 | exon16 | c.G2644A | p.D882N |
| C05 | COL | *OTOF* | exonic | nonsynonymous SNV | NM_194248 | exon15 | c.C1640T | p.T547M |
| C05 | COL | *PMS2* | exonic | nonsynonymous SNV | NM_000535 | exon14 | c.C2395T | p.R799W |
| C05 | COL | *SMPD1* | exonic | nonsynonymous SNV | NM_000543 | exon2 | c.G340A | p.V114M |
| C05 | COL | *TRIOBP* | exonic | nonsynonymous SNV | NM_001039141 | exon19 | c.G6515A | p.R2172Q |
| C05 | COL | *ACADM* | exonic | nonsynonymous SNV | NM_001286043 | exon9 | c.C782A | p.T261N |
| C07 | COL | *CDH1* | exonic | nonsynonymous SNV | NM_004360 | exon16 | c.G2644A | p.D882N |
| C07 | COL | *PMS2* | exonic | nonsynonymous SNV | NM_000535 | exon14 | c.C2395T | p.R799W |
| C111 | COL | *PLEC* | exonic | nonsynonymous SNV | NM_201380 | exon31 | c.C6376T | p.R2126C |
| C113 | COL | *MSH2* | exonic | nonsynonymous SNV | NM_000251 | exon1 | c.T55C | p.F19L |
| C114 | COL | *NBN* | exonic | nonsynonymous SNV | NM_002485 | exon10 | c.A1354C | p.T452P |
| C119 | COL | *BLM* | exonic | nonsynonymous SNV | NM_000057 | exon2 | c.C43T | p.R15C |
| C119 | COL | *C2CD3* | exonic | nonsynonymous SNV | NM_001286577 | exon10 | c.C1615T | p.H539Y |
| C126 | COL | *NF1* | exonic | nonsynonymous SNV | NM_001042492 | exon19 | c.G2294A | p.R765H |
| C130 | COL | *FANCM* | exonic | nonsynonymous SNV | NM_020937 | exon10 | c.C1741T | p.R581C |
| C131 | COL | *OXCT1* | exonic | nonsynonymous SNV | NM_000436 | exon15 | c.G1367T | p.C456F |
| C133 | COL | *FANCM* | exonic | nonsynonymous SNV | NM_020937 | exon10 | c.C1741T | p.R581C |
| C18 | COL | *DSP* | exonic | nonsynonymous SNV | NM_004415 | exon24 | c.C6881G | p.A2294G |
| C18 | COL | *TTN* | exonic | nonsynonymous SNV | NM_001267550 | exon251 | c.C46702T | p.P15568S |
| C19 | COL | *KLF11* | exonic | nonsynonymous SNV | NM_001177718 | exon3 | c.G1118A | p.R373Q |
| C19 | COL | *SPTAN1* | exonic | nonsynonymous SNV | NM_001130438 | exon43 | c.C5545T | p.R1849W |
| C19 | COL | *TTN* | exonic | nonsynonymous SNV | NM_001267550 | exon251 | c.C46847T | p.T15616M |
| C20 | COL | *RAD51C* | exonic | nonsynonymous SNV | NM_058216 | exon3 | c.T492G | p.F164L |
| C20 | COL | *TTN* | exonic | nonsynonymous SNV | NM_001267550 | exon349 | c.G97331C | p.R32444P |
| C23 | COL | *WRN* | exonic | nonsynonymous SNV | NM_000553 | exon17 | c.C1909T | p.R637W |
| C25 | COL | *ALK* | exonic | nonsynonymous SNV | NM_004304 | exon11 | c.G1918A | p.G640R |
| C36 | COL | *ATM* | exonic | nonsynonymous SNV | NM_000051 | exon49 | c.C7174T | p.R2392W |
| C45 | COL | *MSH6* | exonic | nonsynonymous SNV | NM_000179 | exon8 | c.T3758A | p.V1253E |
| C48 | COL | *BRCA2* | exonic | nonsynonymous SNV | NM_000059 | exon11 | c.C3032G | p.T1011R |
| C50 | COL | *BRCA1* | exonic | nonsynonymous SNV | NM_007294 | exon17 | c.C5123A | p.A1708E |
| C56 | COL | *FANCE* | exonic | nonsynonymous SNV | NM_021922 | exon2 | c.C397T | p.L133F |
| C56 | COL | *MSH2* | exonic | nonsynonymous SNV | NM_000251 | exon5 | c.C815T | p.A272V |
| C56 | COL | *PMS2* | exonic | nonsynonymous SNV | NM_000535 | exon14 | c.G2335A | p.G779R |
| C65 | COL | *PMS2* | exonic | nonsynonymous SNV | NM_000535 | exon14 | c.C2395T | p.R799W |
| C65 | COL | *SLX4* | exonic | nonsynonymous SNV | NM_032444 | exon12 | c.G2585A | p.R862Q |
| C70 | COL | *BRCA1* | exonic | nonsynonymous SNV | NM_007294 | exon17 | c.C5123A | p.A1708E |
| C74 | COL | *BRCA1* | exonic | nonsynonymous SNV | NM_007294 | exon17 | c.C5123A | p.A1708E |
| C74 | COL | *WT1* | exonic | nonsynonymous SNV | NM_024426 | exon6 | c.T1048C | p.C350R |
| C80 | COL | *CDKN2A* | exonic | nonsynonymous SNV | NM_058197 | exon1 | c.G104T | p.G35V |
| C84 | COL | *PMS2* | exonic | nonsynonymous SNV | NM_000535 | exon14 | c.C2395T | p.R799W |
| C86 | COL | *PMS2* | exonic | nonsynonymous SNV | NM_000535 | exon14 | c.C2395T | p.R799W |
| C89 | COL | *PMS2* | exonic | nonsynonymous SNV | NM_000535 | exon11 | c.T1271C | p.F424S |
| C95 | COL | *RET* | exonic | nonsynonymous SNV | NM_020975 | exon19 | c.G3149A | p.R1050Q |
| GT260 | MEX | *PTCH2* | exonic | nonsynonymous SNV | NM_003738 | exon3 | c.T395C | p.L132P |
| GT262 | MEX | *CHEK2* | exonic | nonsynonymous SNV | NM_001005735 | exon11 | c.G1153A | p.G385S |
| GT264 | MEX | *MSR1* | exonic | nonsynonymous SNV | NM_138716 | exon9 | c.A1133G | p.H378R |
| GT265 | MEX | *APC* | exonic | nonsynonymous SNV | NM_000038 | exon16 | c.A2023G | p.T675A |
| GT267 | MEX | *RTEL1* | exonic | nonsynonymous SNV | NM_001283009 | exon8 | c.A640T | p.N214Y |
| GT271 | MEX | *APC* | exonic | nonsynonymous SNV | NM_000038 | exon16 | c.C8042T | p.P2681L |
| GT272 | MEX | *SLX4* | exonic | nonsynonymous SNV | NM_032444 | exon2 | c.G421T | p.G141W |
| GT272 | MEX | *RAD50* | exonic | nonsynonymous SNV | NM_005732 | exon4 | c.C545A | p.A182E |
| GT275 | MEX | *BRCA2* | exonic | nonsynonymous SNV | NM_000059 | exon19 | c.C8420T | p.S2807L |
| GT276 | MEX | *FANCM* | exonic | nonsynonymous SNV | NM_020937 | exon4 | c.C808T | p.R270C |
| GT277 | MEX | *KDR* | exonic | nonsynonymous SNV | NM_002253 | exon25 | c.G3353A | p.R1118Q |
| GT282 | MEX | *RET* | exonic | nonsynonymous SNV | NM_020975 | exon19 | c.C3142T | p.L1048F |
| GT284 | MEX | *ATR* | exonic | nonsynonymous SNV | NM_001184 | exon12 | c.C2615T | p.T872I |
| GT292 | MEX | *RSPO1* | exonic | nonsynonymous SNV | NM_001242908 | exon6 | c.G553T | p.G185W |
| GT294 | MEX | *RNASEL* | exonic | nonsynonymous SNV | NM_021133 | exon2 | c.T551C | p.L184S |
| GT298 | MEX | *SLX4* | exonic | nonsynonymous SNV | NM_032444 | exon12 | c.C3782T | p.P1261L |
| GT404 | MEX | *CDKN2A* | exonic | nonsynonymous SNV | NM_058197 | exon1 | c.T146C | p.I49T |
| GT404 | MEX | *FANCM* | exonic | nonsynonymous SNV | NM_020937 | exon10 | c.C1741T | p.R581C |
| TISEM01 | MEX | *BRCA1* | exonic | nonsynonymous SNV | NM_007294 | exon17 | c.C5123A | p.A1708E |
| TISEM02 | MEX | *FANCM* | exonic | nonsynonymous SNV | NM_020937 | exon1 | c.A317G | p.N106S |
| TISEM04 | MEX | *TSC2* | exonic | nonsynonymous SNV | NM_000548 | exon18 | c.G1939A | p.D647N |
| TISEM08 | MEX | *FAH* | exonic | nonsynonymous SNV | NM_000137 | exon6 | c.C484T | p.R162C |
| TISEM10 | MEX | *FAH* | exonic | nonsynonymous SNV | NM_000137 | exon9 | c.A715T | p.I239F |
| TISEM10 | MEX | *MRE11* | exonic | nonsynonymous SNV | NM_005591 | exon13 | c.G1384A | p.V462M |
| TISEM11 | MEX | *RTEL1* | exonic | nonsynonymous SNV | NM_001283009 | exon8 | c.A640T | p.N214Y |
| TISEM12 | MEX | *MUTYH* | exonic | nonsynonymous SNV | NM_001128425 | exon13 | c.C1276T | p.R426C |
| TISEM13 | MEX | *PTEN* | exonic | nonsynonymous SNV | NM_000314 | exon4 | c.A227G | p.Y76C |
| TISEM15 | MEX | *APC* | exonic | nonsynonymous SNV | NM_000038 | exon15 | c.T1895C | p.I632T |
| TISEM17 | MEX | *FAH* | exonic | nonsynonymous SNV | NM_000137 | exon9 | c.A715T | p.I239F |
| TISEM19 | MEX | *SLX4* | exonic | nonsynonymous SNV | NM_032444 | exon10 | c.G2157T | p.Q719H |
