# Supplementary material for: Latin American Study of Hereditary Breast and Ovarian Cancer LACAM: A Genomic Epidemiology Approach
Source: Front Oncol. 2019 Dec 20;9:1429. doi: 10.3389/fonc.2019.01429 (PMC6933010; doi:10.3389/fonc.2019.01429)
Supplement: Supplementary Table 2 — Clinical and epidemiological characteristics of individuals recruited in the first phase of the LACAM study by country. DCIS, Ductal carcinoma in situ; IDC, Invasive ductal carcinoma; ILC, Invasive lobular carcinoma; MC, Medullary carcinoma; TN, Triple negative; SC, Serous carcinoma; EC, Endometrioid carcinoma; CCC, Clear cell carcinoma; (*) Breast, Colon, Thyroid, Pancreas, Endometrial. NA, Not applicable. [file Table_2.DOCX]

**Supplementary Table 2. Clinical and epidemiological characteristics of individuals recruited in the first phase of the *LACAM* study by country.** DCIS: Ductal carcinoma *in situ*, IDC: Invasive ductal carcinoma, ILC:Invasive lobular carcinoma, MC: Medullary carcinoma, TN: Triple negative, SC: Serous carcinoma, EC: Endometrioid carcinoma, CCC: Clear cell carcinoma. (*) Breast, Colon, Thyroid, Pancreas, Endometrial.NA: Not applicable

| **Epidemiological Characteristics (N Total=403)** | | | | | |
| --- | --- | --- | --- | --- | --- |
|  | **Argentina** | **Colombia** | **Guatemala** | **Mexico** | **Peru** |
|  | **n (%)** | **n (%)** | **n (%)** | **n (%)** | **n (%)** |
| **BMI** |  |  |  |  |  |
| Underweight (<18.5) | 4 (7.1) | 2 (1.3) | 0 (0.0) | 0 (0.0) | 2 (3.9) |
| Normal (18.5<25) | 22 (39.3) | 61 (38.1) | 11 (55.0) | 41 (35.3) | 20 (39.2) |
| Overweight (25.0<30) | 16 (28.6) | 49 (30.6) | 5 (25.0) | 39 (33.6) | 19 (37.3) |
| Obese (>30) | 8 (14.3) | 12 (7.5) | 3 (15.0) | 31 (26.7) | 10 (19.6) |
| Missing | 6 (10.7) | 36 (22.5) | 1 (5.0) | 5 (4.3) | 0 (0.0) |
| **Age, y** |  |  |  |  |  |
| 18-45 | 16 (28.6) | 71 (44.4) | 14 (70.0) | 52 (44.8) | 37 (72.5) |
| 46–60 | 21 (37.5) | 63 (39.3) | 5 (25.0) | 58 (50.0) | 13 (25.5) |
| 61–70 | 7 (12.5) | 15 (9.4) | 0 (0.0) | 6 (5.2) | 1 (2.0) |
| ≥71 | 8 (14.3) | 8 (5.0) | 0 (0.0) | 0 (0.0) | 0 (0.0) |
| Missing | 4 (7.1) | 3 (1.9) | 1 (5.0) | 0 (0.0) | 0 (0.0) |
| **Gender** |  |  |  |  |  |
| Male | 0 (0.0) | 3 (1.9) | 0 (0.0) | 6 (5.2) | 0 (0.0) |
| Female | 56 (100) | 157 (98.1) | 20(100) | 110 (94.8) | 51(100) |
| **Race/ethnicity** |  |  |  |  |  |
| White | 54 (96.4) | 25 (15.6) | 1 (5.0) | 34 (29.3) | 5 (9.8) |
| Black | 0 (0.0) | 0 (0.0) | 1 (5.0) | 0 (0.0) | 0 (0.0) |
| Mestizo/Mulatto | 0 (0.0) | 56 (35.0) | 16 (80.0) | 77 (66.4) | 45 (88.2) |
| Asiatic | 1 (1.8) | 0 (0.0) | 0 (0.0) | 0 (0.0) | 0 (0.0) |
| Indigenous | 0 (0.0) | 0 (0.0) | 1 (5.0) | 4 (3.4) | 1 (2.0) |
| Other/unknown | 1 (1.8)) | 79 (49.4) | 1 (5.0) | 1 (0.9) | 0 (0.0) |
| **Education level** |  |  |  |  |  |
| Post graduate | 0 (0.0) | 10 (6.2) | 2 (10.0) | 14 (12.1) | 1 (2.0) |
| Graduate | 30 (53.6) | 23 (14.4) | 2 (10.0) | 28 (24.1) | 6 (11.7) |
| Superior-Technical | 18 (32.1) | 19 (11.9) | 7 (35.0) | 28 (24.1) | 8 (15.7) |
| Secondary | 5 (8.9) | 25 (15.6) | 2 (10.0) | 18 (15.5) | 25 (49.0) |
| Primary | 0 (0.0) | 4 (2.5) | 6 (30.) | 25 (21.6) | 10 (19.6) |
| None | 0 (0.0) | 0 (0.0) | 0 (0.0) | 2 (1.7) | 1 (2.0) |
| No information | 3 (5.4) | 79 (49.4) | 1(5.0) | 1 (0.9) | 0 (0.0) |
| **Smoking history** |  |  |  |  |  |
| Never smoker | 44 (78.6) | 59 (36.9) | 18 (90.0) | 81 (69.3) | 50 (98.0) |
| Former smoker | 8 (14.3) | 21 (13.1) | 1 (5.0) | 29 (25.0) | 1 (2.0) |
| Current smoker | 3 (5.4) | 1 (0.6) | 0 (0.0) | 6 (5.2) | 0 (0.0) |
| No Information | 1 (1.8) | 79 (49.4) | 1 (5.0) | 0 (0.0) | 0 (0.0) |
| **Alcohol history** |  |  |  |  |  |
| Never drinker | 55 (98.2) | 67 (41.9) | 15 (75.0) | 76 (65.5) | 50 (98.0) |
| Former drinker | 0 (0.0) | 12 (7.5) | 3 (15.0) | 18 (15.5) | 1 (2.0) |
| Current drinker | 0 (0.0) | 2 (1.2) | 1 (5.0) | 22 (19.0) | 0 (0.0) |
| No Information | 1 (1.8) | 79 (49.4) | 1 (5.0) | 0 (0.0) | 0 (0.0) |
| **Family history of cancer** |  |  |  |  |  |
| Yes | 53 (94.6) | 115 (71.9) | 16 (80.0) | 21 (18.1) | 25 (49.0) |
| No | 2 (3.6) | 44 (27.5) | 3 (15.0) | 15 (12.9) | 26 (51.0) |
| Missing | 1 (0.8) | 1 (0.6) | 1 (5.0) | 80 (69.0) | 0 (0.0) |
| **Pregnancy** |  |  |  |  |  |
| Yes | 45 (80.4) | 100 (62.5) | 14 (70.0) | 90 (77.6) | 43 (84.3) |
| No | 10 (17.9) | 38 (23.8) | 5 (25.0) | 20 (17.2) | 8 (15.7) |
| Missing | 1 (1.8) | 22 (13.8) | 1 (5.0) | 6 (5.2) | 0 (0.0) |
| **Clinical Characteristics (N=356)** | | | | | |
| **Primary cancer site** |  |  |  |  |  |
| Breast | 39 (83.0) | 105 (82.0) | 16 (88.8) | 58 (51.3) | 50 (100) |
| Ovary | 6 (12.8) | 20 (15.6) | 1 (5.6) | 48(42.5) | 0 (0.0) |
| Multiple primaries (*) | 2 (4.2) | 2 (1.6) | 0 (0.0) | 0 (0.0) | 0 (0.0) |
| Missing | 0 (0.0) | 1 (0.8) | 1 (5.6) | 7 (6.2) | 0 (0.0) |
| **Histopathological subtype Breast cancer** |  |  |  |  |  |
| DCIS | 0 (0.0) | 0 (0.0) | 0 (0.0) | 1 (1.7) | 0 (0.0) |
| IDC | 26 (66.7) | 60 (57.1) | 0 (0.0) | 35 (60.3) | 48 (96.0) |
| ILC | 5 (12.8) | 6 (5.7) | 4 (25.0) | 2 (3.5) | 0 (0.0) |
| MC | 0 (0.0) | 1 (0.9) | 0 (0.0) | 0 (0.0) | 0 (0.0) |
| TN | 0 (0.0) | 0 (0.0) | 0 (0.0) | 8 (13.8) | 0 (0.0) |
| Missing | 8 (20.5) | 38 (36.2) | 12 (75.0) | 12 (20.7) | 2 (4.0) |
| **Histopathological subtype Ovarian cancer** |  |  |  |  |  |
| SC | 6 (100) | 5 (25.0) | 1 (100) | 34 (70.8) | NA |
| EC | 0 (0.0) | 0 (0.0) | 0 (0.0) | 5 (10.4) | NA |
| CCC | 0 (0.0) | 3 (15.0) | 0 (0.0) | 0 (0.0) | NA |
| Missing | 0 (0.0) | 12 (60.0) | 0 (0.0) | 9 (18.8) | NA |
| **Stage at diagnosis** |  |  |  |  |  |
| I | 0 (0.0) | 12 (7.5) | 0 (0.0) | 10 (8.6) | 3 (5.9) |
| II | 0 (0.0) | 28 (17.5) | 5 (25.0) | 15 (12.9) | 5 (9.8) |
| III | 0 (0.0) | 15 (9.4) | 8 (40.0) | 34 (29.3) | 11 (21.6) |
| IV | 0 (0.0) | 2 (1.2) | 0 (0.0) | 2 (1.7) | 0 (0.0) |
| No Information | 56 (100) | 103 (64.4) | 7 (35.0) | 55 (47.5) | 32 (62.7) |
